# Clinical and ultrasound characteristics of pediatric lateral neck masses

**DOI:** 10.1371/journal.pone.0251563

**Published:** 2021-05-12

**Authors:** Nemanja Rankovic, Jovana Todorovic, Radoje Simic

**Affiliations:** 1 Department of Plastic and Reconstructive Surgery, Institute of Mother and Child Health Care of Serbia "Dr Vukan Cupic", Belgrade, Serbia; 2 Institute of Social Medicine, Faculty of Medicine, University of Belgrade, Belgrade, Serbia; 3 Department of Surgery, Faculty of Medicine, University of Belgrade, Belgrade, Serbia; University of Porto Faculty of Medicine, PORTUGAL

## Abstract

Lateral neck masses (LNM) often present a diagnostic challenge in the practice of pediatric plastic surgeon. The aim of this study is to investigate the clinical and ultrasound (US) characteristics of pediatric LNM in order to make mutual comparison between their entities and enable the most accurate preoperative diagnosis. A cross-sectional study was conducted among 250 pediatric patients treated by surgical excision or sclerotherapy in our institution in the period from July 2009 to June 2019. Lymphatic malformation was the most frequent congenital LNM (60.9%), while reactive or granulomatous lymphadenitis was the most frequent acquired LNM (47%). Congenital anomalies were significantly more often localized in the upper half of the sternocleidomastoid (SCM) muscle region, and had more often soft consistency than acquired ones. Congenital LNM had a 32.37 (3.44–304.63) times higher likelihood of incorrect (p = 0.002) and 5.86 (1.35–25.48) times higher likelihood of undetermined (p = 0.018) than correct US findings, respectively. Acquired LNM were significantly more often localized in the region behind the SCM muscle and more often had solid US appearance in comparison to the congenital ones. Association of the clinical and US findings is very important in determining the most accurate preoperative diagnosis without exposing the children to unnecessary utilizing ionizing radiation or anesthesia. Although they are mostly benign, extreme caution is necessary due to malignancies which were found in 16.4% of all our patients.

## Introduction

Lateral neck masses (LNM) are common in pediatric population. Although they are mostly benign, extreme caution is necessary because malignancies are diagnosed in about 12% to 15% of all neck masses in children [1, 2]. Knowledge of embryology, anatomy, and clinical presentation currently helps establishing the surgeon’s preoperative diagnosis. Medical history and physical examination are the first steps, but the additional radiological analyses are often required [3]. Clinical and ultrasound (US) findings are very important in making differential diagnosis and, in many cases, determine the most likely cause of neck swelling. Without utilizing ionizing radiation, iodinated contrast material, sedation and/or anesthesia, US provides tool for quick and cost-effective acquisition of information, including the localization, size, shape, internal content, vascularity of the mass and its relationship with surrounding anatomical structures. The goal of US is often to determine the next best step, including clinical observation, follow-up US, radiography, magnetic resonance imaging (MRI), computed tomography scan (CT), biopsy, surgical excision, embolization and sclerosation [4]. While US is a standard diagnostic procedure, CT and/or MRI are still the most commonly used imaging methods for neck masses by some practitioners [5, 6]. Accurate preoperative diagnosis is crucial in order to enable adequate operative technique and avoid consequent intra- and postoperative complications and relapses [7]. Excision is preferred surgical treatment in most neck anomalies, but sclerotherapy is the main option for the treatment of lymphatic malformations (LM) [8, 9]. Namely, complete excision of LMs is usually not possible due to the infiltrative nature of the lesion, and the rate of clinically significant recurrence has been reported to be as high as 40% [7]. However, some authors reported that spontaneous regression of LMs was seen in 11.4% to 12.5% of patients [10, 11]. Difficulty to establish the accurate preoperative diagnosis of pediatric neck masses, despite using US, MRI and CT, was shown in one of the few studies in which histopathological examination has confirmed it in only 58% [3]. To the best of our knowledge, there are no published large series of pediatric LNM in which clinical and US characteristics are examined thoroughly.

The aim of this study is to investigate the clinical and US characteristics of pediatric LNM, in order to make mutual comparison between their entities and enable the most accurate preoperative diagnosis.

## Material and methods

A cross-sectional study was conducted, including all patients with LNM who were presented to the Department of Plastic and Reconstructive Surgery, Institute of Mother and Child Health Care of Serbia "Dr Vukan Cupic" in Belgrade, Serbia. The study included all the patients treated by surgical excision or sclerotherapy in this institution from July 2009 to June 2019. Two hundred and fifty patients were included in the analysis.

The patients’ data were taken from their medical histories. They included demographic characteristics (sex, age); localization of the neck mass: upper half of the sternocleidomastoid (SCM) muscle region (includes parotid region as well as upper medial and lateral part of the SCM muscle with upper half of its anterior border), lower half of the SCM muscle region (includes lower medial and lateral part of the SCM muscle with lower half of its anterior border), region behind the SCM muscle (it is "true" lateral cervical region that includes supraclavicular region); its consistency on physical examination (soft, hard and moderately hard); US neck mass appearance (non-solid or cystic and solid); US diagnosis (correct, incorrect and undetermined), as well as children laboratory analyses (white blood cell count—WBC and C-reactive protein—CRP).

The duration of periods between the onset of the LNM and performing an operation was divided as following: less than 4 weeks, from 4 to 8 weeks or more than 8 weeks.

The LNM were classified in two groups: congenital (which included: branchial cleft cyst (BCC), bronchogenic cyst, venous and lymphatic malformations (VM, LM, respectively), dermoid cyst, ganglioneuroma, ganglioneuroblastoma, neuroblastoma, neurofibromatosis and congenital rhabdomyosarcoma) and acquired (reactive or granulomatous lymphadenitis, pilomatrixoma, lymphoma, lipoblastoma, rhabdomyosarcoma, Langerhans cell histiocytosis and acinic cell carcinoma of salivary gland).

In order for an anomaly to be declared congenital, there must be a residual embryonic structure or an embryonic disorder that will be clinically presented as pathological in a certain period of intrauterine or postnatal life. There is another point of view concerning congenital tumors. For a tumor to be declared congenital, it must be clinically or radiologically presented at birth or in the first four weeks of life. According to some authors, this period can be extended to three and even twelve months of age [12–14].

Some neck masses (neurofibromatosis, LM, lymphadenitis, lymphomas) are often presented in several regions, on one or both sides of the neck and, in our study, the localization is determined according to the region where the mass was firstly noticed or where the swelling was the most prominent.

We did not have any patient with first branchial cleft cyst, yet children with this type of anomaly were presented to us with sinus or fistula. Third and fourth branchial cleft cysts were located near the left lobe of the thyroid gland in all of our patients, and we attributed them to the anterior region of the neck.

This study was approved by the Ethics Committee of the University of Belgrade—Faculty of -Medicine (decision number—1550/V-37). Written informed consent was obtained from parents, as well as from patients if they were over fifteen years old.

## Statistical analysis

As far as descriptive statistics parameters are concerned, mean value, standard deviation, minimum and maximum value, median and interquartile range (IQR) were used.

The differences in clinical and US characteristics of LNM groups were examined using the Mann-Whitney U test (continuous variables) and Chi-square test (categorical variables).

Binary logistic regression analysis was started with univariate analysis, with the congenital LNM as an dependent (target) variable and all other clinical and US parameters as independent variables (sex, age, US diagnosis, localization, neck mass side, consistency, US appearance, symptoms, laboratory analyses and duration of periods between the onset of the lateral neck mass and the operation). Variables which were found significant at 5% level in univariate analysis further entered in the multiple logistic regression analysis (method: Enter).

The adequacy of the model was further checked with Hosmer-Lemeshaw goodness-of-fit test.

Throughout the study, statistical significance was assessed at the 5% level.

All analyses were done using the Statistical Package for Social Science (SPSS) 22.0.

Sensitivity analysis for diagnostic methods used in order to establish diagnoses (US diagnoses and clinical diagnoses, such as consistency of the neck masses) has been performed. It was based on 2 × 2 contingency table. Sensitivity has been obtained according to the following formula:

$$\underline{TP}\;X\;100=Sensitivity\;\left(\%\right),\;TP+FN$$

Where: TP denotes True Positive, while FN denotes False Negative values.

Analysis has been performed by using Microsoft Excel 2010.

## Results

The demographic, clinical, and US characteristics of lateral neck masses in children are presented in Table 1.

**Table 1:** The demographic, clinical, and ultrasound characteristics of pediatric patients with congenital and acquired lateral neck masses.

Parameters	Congenital N (%)	Acquired N (%)	p-value
**Sex**			
Male	67 (50.4)	56 (47.9)	
Female	66 (49.6)	61 (52.1)	0.787
**Age (months), median (IQR)**^**1**^	60.0 (18.0–136.0)	108.0 (61.5–175.0)	0.001*
**Ultrasound diagnosis**			
Correct	97 (72.9)	112 (95.7)	
Incorrect	17 (12.8)	1 (0.9)	
Undetermined	19 (14.3)	4 (3.4)	<0.001**
**Localization**			
**Upper half of SCM muscle region**	**70 (52.6)**	**33 (28.2)**	
**Lower half of SCM muscle region**	**19 (14.3)**	**19 (16.2)**	
**Region behind SCM muscle**	**44 (33.1)**	**65 (55.6)**	<0.001**
**Neck mass side**			
Left	73 (54.9)	55 (47.0)	
Right	58 (43.6)	53 (45.3)	0.045**
Bilateral	2 (1.5)	9 (7.7)	
**Consistency**			
Soft	**90 (67.7)**	4 (3.4)	
Hard	**23 (17.3)**	74 (63.2)	
Moderately hard	**20 (15.0)**	39 (33.3)	<0.001**
**Ultrasound apperance**			
Non-solid (cystic)	72 (54.1)	1 (0.9)	
Solid	61 (45.9)	116 (99.1)	<0.001**
**Symptoms**			
Yes	28 (21.1)	66 (56.4)	
No	105 (78.9)	51 (43.6)	0.002**
**Laboratory analyses**			
Normal	122 (91.7)	112 (95.7)	
Outside referent values	11 (8.3)	5 (4.3)	0.303
**Duration of periods between the onset of the lateral neck mass and the operation**			
< 4 weeks	60 (45.1)	40 (34.2)	
4–8 weeks	22 (16.5)	40 (34.2)	
> 8 weeks	51 (38.3)	37 (31.6)	0.005**

^1^IQR (interquartile range)

*Mann-Whitney U test

** Chi-square test; SCM—sternocleidomastoid

The study included total of 250 pediatric patients with LNM. More than a half of them (133 patients or 53.2%) had congenital, while 117 (46.8%) had acquired LNM (Table 1).

One hundred and twenty-seven patients were girls (50.80%), while one hundred and twenty-three were boys (49,2%).

Patients with acquired LNM were significantly older [108.0 (61.5–175.0) *vs*. 60.0 (18.0–136.0), p<0.001]; (Table 1).

The most frequent congenital LNM were LM (81 patient) and BCC (26 patients), while the most frequent acquired LNM were reactive or granulomatous lymphadenitis (55 patients), Hodgkin lymphoma (24 patients) and pilomatrixoma (23 patients) (Table 2). Two hundred and nine patients had benign neck mass, while forty-one (16.4%) had malignancy. Results of histopathology and cytology examinations in patients with LNM are presented in Table 2.

**Table 2:** Findings of histopathology and citology examinations in pediatric patients with lateral neck masses.

a. Congenital LNM		b. Acquired LNM	
	N (%)		N (%)
Congenital LNM	133 (100)	Acquired LNM	117 (100)
Lymphatic malformations	81 (60.9)	Reactive or Granulomatous lymphadenitis	55 (47.00)
Branchial cleft cyst	26 (19.55)	Hodgkin lymphoma	24 (20.50)
Dermoid cyst	13 (9.77)	Pilomatrixoma	23 (19.66)
Venous malformations	5 (3.76)	Burkitt lymphoma	5 (4.27)
Neurofibromatosis	2 (1.50)	Langerhans cell histiocytosis	3 (2.56)
Ganglioneuroma	2 (1.50)	T lymphoma	2 (1.70)
Bronchogenic cyst	1 (0.75)	Acinic cell carcinoma	2 (1.70)
Neuroblastoma	1 (0.75)	Rhabdomyosarcoma	2 (1.70)
Ganglioneuroblastoma	1 (0.75)	Lipoblastoma	1 (0.85)
Congenital rhabdomyosarcoma	1 (0.75)		

The correct US diagnosis was present in significantly higher percentages in a group with acquired LNM. Considering localization of the neck tumors, congenital LNM appeared significantly more often in upper half of SCM muscle region, while acquired ones were significantly more often in the region behind SCM muscle (Table 1). There were no differences between the neck side presentation of congenital and acquired masses except bilaterally appearing ones, which were significantly more often acquired tumors and diseases (Table 1). Congenital LNM had soft consistency in 67.7%, hard in 17.3% and moderately hard consistency in 15.0%, while acquired LNM had soft, hard and moderately hard consistency in 3.4%, 63.2% and 33.3%, respectively (Table 1). Comparing these three categories, there was significant difference between the two groups; congenital anomalies had highly significant more often soft consistency than acquired tumors. Acquired LNM had solid US appearance significantly more often than congenital ones.

Absence of symptoms was statistically significant more often in congenital tumors. There were no differences between the two groups of LNM considering laboratory analyses, with highly dominant normal values in both groups. Duration of the period between the onset of the LNM and performing an operation was significantly different between two groups, but most prominently when it lasted between 4 and 8 weeks in acquired tumors in comparison to congenital ones (34.2% *vs* 16.5%, respectively).

Using univariate logistic regression analysis, a statistically significant association of age, US diagnosis, localization, consistency, symptoms and duration of periods between the onset of the lateral neck mass and the operation, with congenital LNM, was determined. Therefore, all these independent parameters which were found significant at 5% level in univariate analysis were further used in the model of multiple regression analysis.

Model adequacy of multiple logistic regression analysis, with congenital LNM as an outcome variable and independent variables mentioned above, was verified by using the Hosmer and Lemeshow Test.

The application of this test showed (χ^2^ = 12.154; p = 0.144) that the selected predictors were appropriate and suitable for performing this analysis.

**Fig 1:**
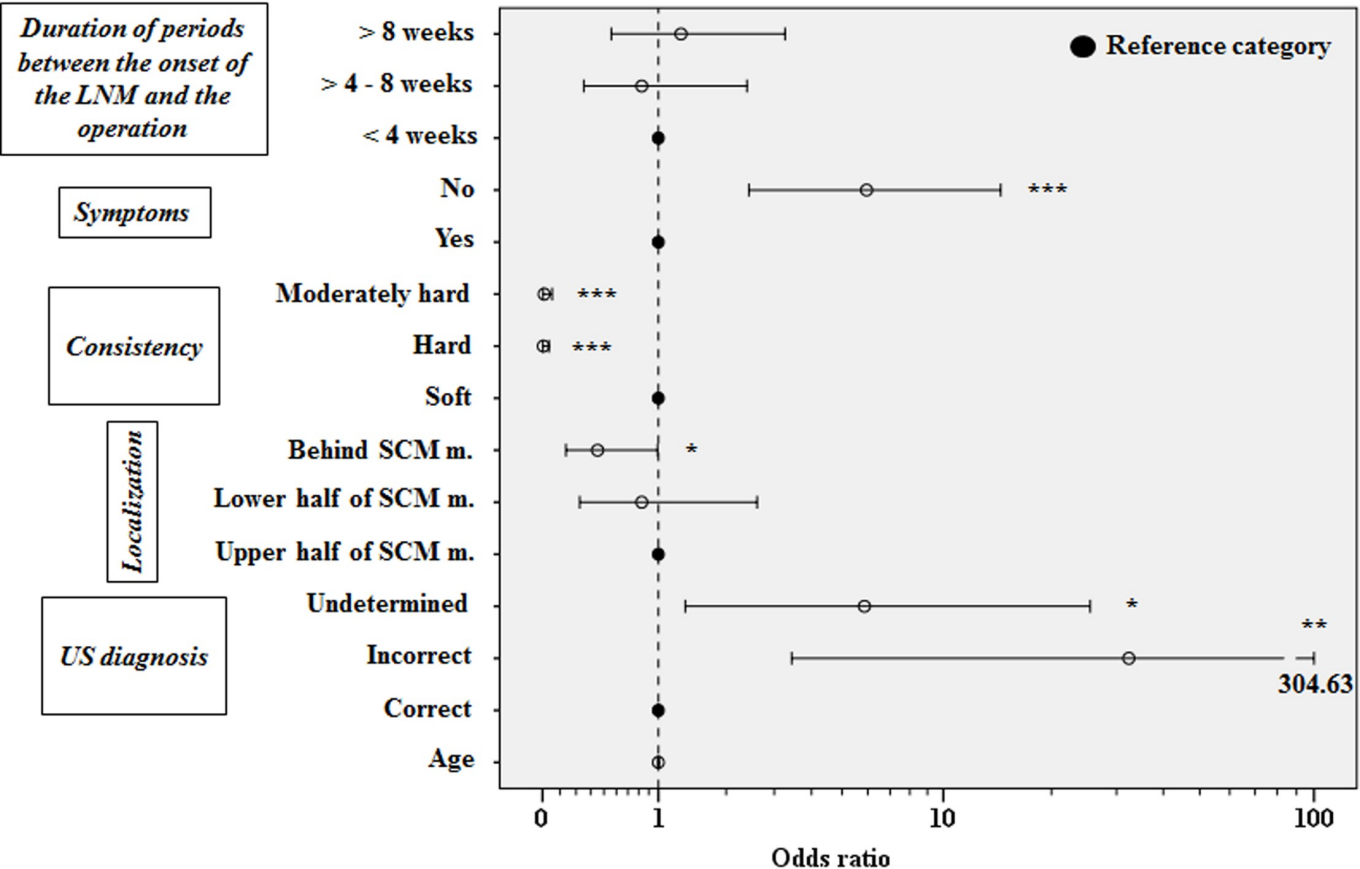
The results of multiple logistic regression analysis with congenital lateral neck masses as an outcome variable. * p < 0.05; ** p < 0.01; *** **p < 0.001** *vs* corresponding reference category; dotted line indicates value of equal chances for manifestation of selected clinical and US parameters.

Multiple logistic regression analysis, with congenital LNM as an outcome variable, showed that patients with congenital LNM had 32.37 (3.44–304.63) times higher likelihood of incorrect than correct US findings (p = 0.002) (Fig 1).

**Fig 2:**
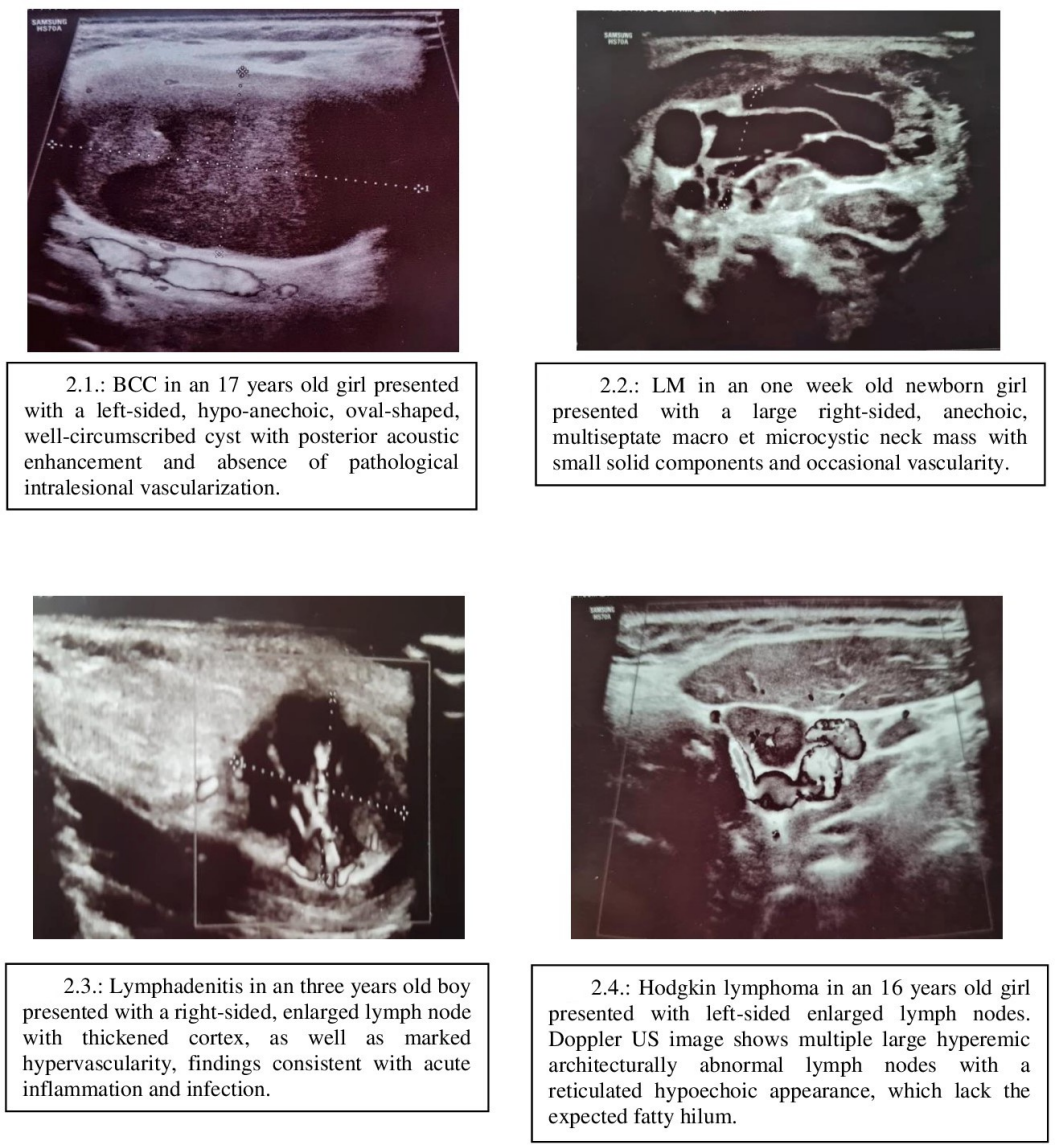
Images of lateral neck masses ultrasound findings (BCC-branchial cleft cyst; LM-lymphatic malformations; US-ultrasound).

Moreover, patients with congenital neck tumors have 5.86 (1.35–25.48) times higher likelihood to have undetermined US findings compared to the probability of the correct findings (p = 0.018) (Fig 1). US characteristics of our most frequent LNM are presented in Fig 2.

**Fig 3:**
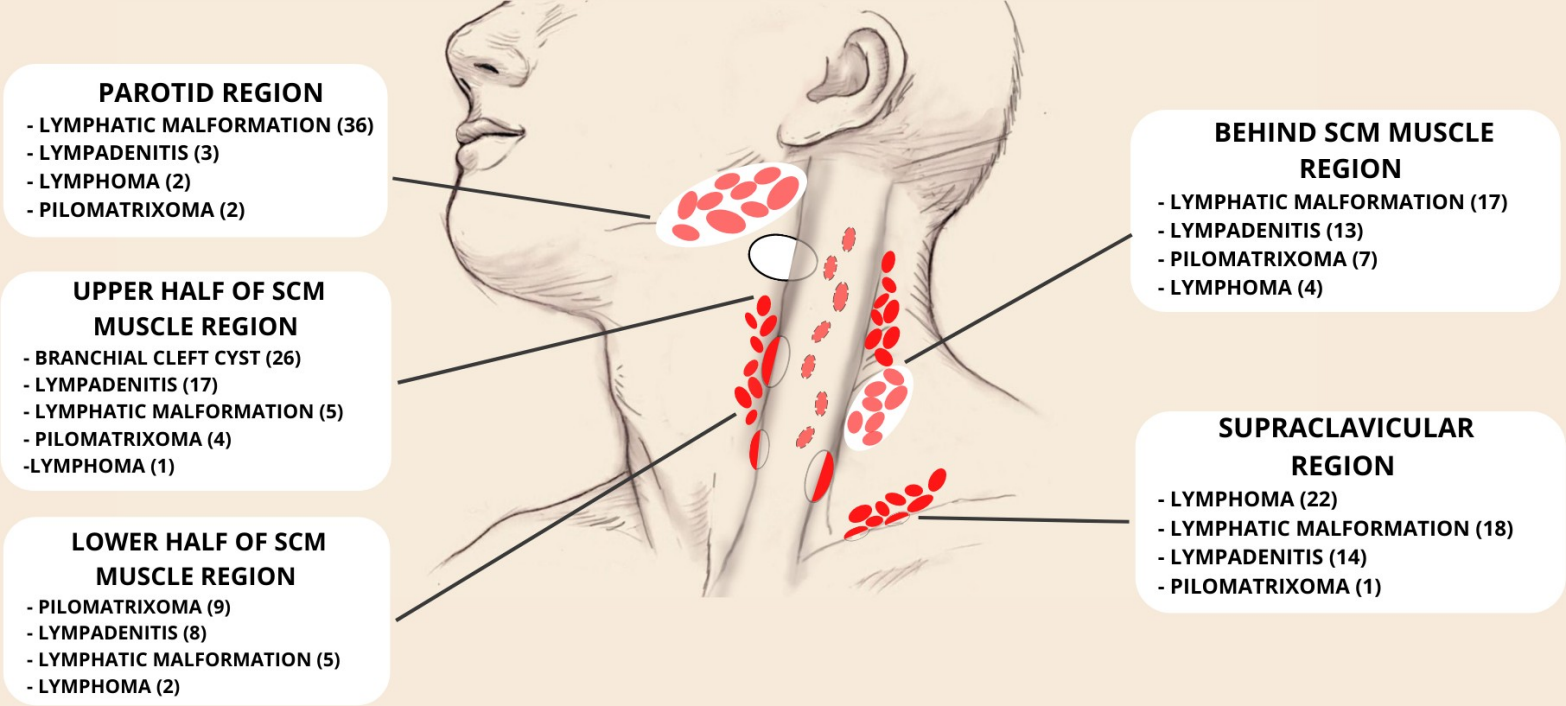
Anatomical scheme of lateral neck masses localization in our study (SCM—sternocleidomastoid).

The localization behind the SCM muscle had 2.56 (1.01–6.66) times smaller chance of finding LNM in comparison to a localization of upper half of SCM muscle in a group of congenital anomalies (p = 0.048). Region of lower half of SCM muscle is 1.23 (0.38–4.00) times less likely to be a localization of congenital neck tumors than the ones situated at the upper half of SCM muscle but this relation did not reach statistical significance (p = 0.721) (Figs 1 and 3).

When considering the consistency of LNM in children, congenital tumors were 102 (25.0–333.33; p = 0.001) and 99 (16.66–250.0; p = 0.001) times less likely to have hard and moderately hard consistency in comparison to soft one, respectively.

Regarding the symptoms that accompany these anomalies, congenital LNM were 5.95 (2.44–14.51) times more likely to be asymptomatic than symptomatic (p = 0.001) (Fig 1). Neither of the established and compared periods between the onset of the LNM and performing an operation were significantly associated with the congenital LNM, according to multiple logistic regression analysis (Fig 1).

The strength of the study and the required number of patients in the groups (congenital and acquired lateral neck masses) were not calculated before the start of the study. At the end of the study, considering the objectives of the study, we selected a couple of key parameters (US diagnosis—% of correct findings in both groups; US apperance—% of solid findings in both groups) that are shown in Table 1. Subsequently, using those results, we calculated the strength of the study regarding selected parameters and the number of enrolled patients.

In the case of US diagnosis (α err. prob. = 0.05) the study power is 0.9827 and in the case of US appearance (α err. prob. = 0.05) the study power is 1.0000 (in both cases; n = 133; n = 117). Therefore, it seems that the number of patients included in the study is adequate for a reliable assessment of the clinical efficacy of US diagnostics.

The calculations were performed using the G * Power 3.1.3 program (Proportions: Inequality, two independent groups).

In order to get more practical implications for clinicians, sensitivity analysis for diagnostic methods used in order to establish diagnoses (US diagnoses and clinical diagnostic criteria, such as consistency of the neck masses) has been performed (Table 3). The greatest sensitivity was shown for US diagnosis named "correct" (the gold standard was the results of the histopathology report) (72%), as well as for clinical findings in physical examination, specificaly "soft consistency" of the neck mass (66.9%) when the diagnosis of congenital LNM is concerned. On the other hand, in acquired LNM, US diagnoses "correct" showed even higher sensitivity in comparison to congenital LNM (95.7%), while US detected appearance of the lateral neck masses specified as "solid" had sensitivity value as much as 99.1%. In this way, it has been shown that US diagnostics, as a method, with great certainty can indicate LNM, as a diagnosis, as well as type of lesion, congenital or acquired.

**Table 3:** Values of sensitivity for ultrasound and clinical examinations of congenital and acquired lateral neck masses in children.

Sensitivity:	Congenital LNM (%)	Acquired LNM (%)
US diagnosis- correct	72.9	95.7
Consistency of the neck mass- soft	66.9	3.5
Consistency of the neck mass- hard	17.3	63.2
Consistency of the neck mass- moderately hard	15.8	33.3
US apperance of the neck mass—non-solid	54.1	0.8
US apperance of the neck mass—solid	45.9	99.1

LNM- lateral neck masses; US—ultrasound

## Discussion

There are a few studies that investigate LNM in adult population, while, to the best of our knowledge, there is no large study that investigates LNM in pediatric population. Authors Riva et al. reported a monocentric study with 190 pediatric patients with neck masses localized in all regions of the neck [15]. The three largest studies investigating LNM in adult population included data from 135 patients and were monocentric [16, 17], and multicentric [18]. Therefore, this study presents the largest monocentric patient cohort to date, involving 250 children who underwent surgical treatment or sclerotherapy for a LNM.

In the evaluation of LNM, a detailed medical history and physical examination are essential. Laboratory analyses were done in all our patients, as well, out of which only 16 had laboratory findings outside the normal range (8.3% with congenital and 4.3% with acquired LNM). The small number of patients with laboratory results outside the referent values can be explained by the fact that children with neck swellings initially go to the primary care pediatrician who, if suspects on inflammatory enlarged lymph nodes, usually prescribes antibiotics. The majority of children had had the antibiotic therapy prior to the referral to our hospital.

More than a half of our patients, 53.2%, had congenital LNM, while 46.8% had acquired LNM. This is in accordance with the previous studies, as the prevalence of congenital masses varies between 26% and 56%, while the prevalence of acquired masses was between 32% and 48% in different case series [3, 19, 20]. Mean age of the patients with LNM (both congenital and acquired) in our study was similar to those reported in other studies [3, 13, 21]. It was shown that patients with acquired LNM were significantly older than the ones with congenital tumors, while according to multiple logistic regression analysis with congenital LNM as an outcome variable, there was no difference considering the age of the patient. Some authors reported that 80% to 90% of LMs are presented within the first two years of life [22, 23].

In our study, children did not have any symptoms in 62.4%. Multiple logistic regression analysis performed indicated that congenital LNM were 5.95 (2.44–14.51) times more likely to be asymptomatic than symptomatic (p = 0.001). LMs and BCCs, the most common congenital LNM, are typically asymptomatic unless they are under infection and, also, mainly due to their size and localization, when they lead to compression of the surrounding structures [24–28].

As far as US diagnosis is concerned, the difference between congenital and acquired LNM was significant, especially related to correct diagnosis which was present in significantly higher percentages in a group with acquired LNM (95,7%) that mostly included enlarged lymph nodes due to the infection or tumor. US shows an enlarged lymph node as a tumor, but additional examinations, including histopatology, are essential to determine the precise type of the tumor.

Multiple logistic regression analysis indicated that patients with congenital LNM had 32.37 (3.44–304.63; (p = 0.002) times higher likelihood of an incorrect findings, and 5.86 (1.35–25.48; p = 0.018) times higher likelihood of an undetermined findings compared to the probability of correct ones, respectively. Previous studies showed that it can be quite difficult to establish the correct US diagnosis of the congenital neck masses, especially in those that have a variable US presentations, like LMs and BCCs [5, 29–31]. The fact is that it is often difficult to differentiate BCCs from enlarged lymph nodes. For example, authors Gov-Ari et al. showed that preoperative US accuracy for nodal inflammation and congenital lesions were 50.0% and 80.8%, respectively [3]. However, the statistically significant difference between acquired and congenital LNM related the correctness of the US diagnosis in favor of the former one, was confirmed by the finding that the value of sensitivity of this type of examination in acquired neck masses was as high as 95.7%.

Acquired LNM had solid US appearance significantly more frequently than congenital ones, and it was substantiated by the high sensitivity of the US as diagnostic examination. It was previously reported that high percentage of LNM in children (45%) were solid [1, 32].

When considering the consistency of LNM, taking into account all three observed categories, there was significant difference between the two groups, on the account of soft consistency. Namely, congenital anomalies had highly significant more often soft consistency than acquired tumors. This finding was substantiated by multiple logistic regression analysis, according to which congenital tumors were 102 (25.0–333.33; p = 0.001) and 99 (16.66–250.0; p = 0.001) times less likely to be hard and moderately hard, respectively, in comparison to soft consistency.

Actually, congenital neck masses are mostly soft, except when their consistency is changed due to the infection or rapid enlargement. Lymph nodes have particularly hard or moderately hard consistency, except when they have undergone the suppuration process [33, 34]. In our study, acquired LNM in 63.2% and 33.3% had hard and moderately hard consistency, respectively, mostly due to the enlarged lymph nodes. Some anomalies like pilomatrixomas are always firm, stone-like masses [35, 36].

We consider that localization is crucial for determining adequate preoperative diagnosis. Upper half of SCM muscle region (including parotid region as well as upper medial and lateral part of the SCM muscle with its upper half anterior border) is common sites for congenital malformations (patients with LM and BCC) [23, 37, 38]. The most common congenital anomaly, LMs, were most often localized in parotid region (36 patients) while acquired ones were mostly situated in supraclavicular region (22 patients with lymphomas).

In accordance with the opinion of the other authors and based on our experience, supraclavicular neck swelling, presented in children younger than five, are mostly LMs. Moreover, it is known that they often affect several regions of the neck [22, 23]. After fifth year of age, enlarged lymph nodes, especially lymphomas, are frequently localized supraclavicularly [39–41].

To our mind, in addition to the localization, the extent of the swelling, depending on the patients’ age, indicates the accurate diagnosis. Diffuse, soft consistency swelling in preschool children usually indicates LM. At the same age, hard or moderately hard neck swelling particularly refers to an enlarged lymph node. In children older than five, especially at puberty, harder consistency of neck swelling in several regions suggests a possibility of lymphoma [39–41].

Four of our patients were hospitalized in the Oncology department due to the lateral neck swelling, assuming that they had lymph node malignancy, while detailed examination showed that it was BCC, and finally assigned to the group of congenital LNM.

Forty-one of all our patients presented with LNM (16,4%) had malignancies, thirty-one having lymph node malignancy, while ten had tumors of other origin (Table 2). Other studies report malignancies in 12% to 15% of all neck masses [1, 2]. In general pediatric population examined by primary care providers due to persistent lymph node enlargement, surgical biopsy showed malignancy in 15% to 22% [41, 42]. In our study, 86/250 (34,4%) patients with LNM had undergone cervical lymph node biopsy. Fifty-five of them (63.95%), had benign cervical reactive or granulomatous lymphadenitis, while thirty-one (36.05%) showed malignancy (Hodgkin, Burkitt and T lymphoma). Hodgkin lymphoma was the most common malignant tumor of the lateral neck in our study with 58.54% of all malignant tumors.

Forty percent of all our patients were surgically treated within 4 weeks of LNM presentation. Forty-five percent of patients with congenital neck masses were addressed to the pediatric plastic surgeon, examined and operated in the first month of presentation. However, neither of the established and compared periods between the onset of LNM and performing the operation were not significantly associated with the congenital LNM, according to multiple logistic regression analysis. Significant difference between acquired and congenital LNM existed, but was most prominent when period between 4 and 8 weeks was tested (34.2% *vs* 16,5%, respectively). Patients with obvious asymptomatic benign lesions, diagnosed clinicaly and with US, had elective operations sometimes even 8 weeks after their onset. Unfortunately, some parents do not come on time with their children for the treatment, and it also should be said that sometimes they are not sent on time by other doctors working with children.

Finally, we should emphasize the importance of accurate preoperative diagnosis necessary for a surgeon to choose the adequate operative technique. Published reports have shown that sclerotherapy is nearly four times more successful than surgical excision for treating LM and with lower morbidity [43–46].

One hundred seventy-three of our patients had surgical excision (69,2%), while seventy-seven (31,8%) was subjected to sclerotherapy (77 of 81 patients with LM was as much as 95.06%). We did not have any spontaneous regression of the LM reported by other authors [10, 11, 47].

## Conclusion

Association of clinical and US findings is very important in determining the most accurate preoperative diagnosis without exposing the children to unnecessary utilizing ionizing radiation or anesthesia. It has been shown that US diagnostics, as a method, with great certainty can indicate LNM, as a diagnosis, as well as type of lesion, congenital or acquired. Some of the most common congenital LNM encountered in pediatric population are LM and BCC, while the most often acquired tumors are reactive or granulomatous lymphadenitis, Hodgkin lymphomas and pilomatrixomas. Congenital anomalies were significantly more often localized in the upper half of the sternocleidomastoid (SCM) muscle, and had more often soft consistency than acquired ones. Congenital LNM had a 32.37 (3.44–304.63) times higher likelihood of incorrect (p = 0.002) and 5.86 (1.35–25.48) times higher likelihood of undetermined (p = 0.018) than correct US findings, respectively. Acquired LNM were significantly more often localized in the region behind the SCM muscle and more often had solid US appearance in comparison to the congenital ones. Although they are mostly benign, extreme caution is necessary due to malignancies which were found in 16.4% of all our patients.

### Study limitations

The main limitation of our study was that it examined the association of clinical and US characteristics of congenital neck masses in comparison to acquired neck tumors, while differences concerning various histopathological and cytological diagnoses were just descriptive. The study was retrospective, it had a cross-sectional design, so we could not establish the causal relationship between the variables. However, so far there are no published large series of pediatric LNM like ours in which clinical and US characteristics were examined thoroughly.

## Supporting information

S1 Table Full data set for data presented in this study. (XLSX)
